# Assessing work-related musculoskeletal disorders and psychosocial risks in bus drivers: insights from a municipal company case study in Portugal

**DOI:** 10.3389/fpubh.2025.1529023

**Published:** 2025-04-30

**Authors:** Tânia T. Silva, Tatiana R. Mendes, Inês Lapa, Paulo Carvalho, Matilde A. Rodrigues

**Affiliations:** ^1^RISE-Health, Center for Translational Health and Medical Biotechnology Research (TBIO), E2S, Polytechnic of Porto, Porto, Portugal; ^2^Department of Environmental Health, E2S, Polytechnic Institute of Porto, Porto, Portugal

**Keywords:** anxiety, burnout, bus driver, depression, stress, transport sector, WMSD

## Abstract

**Introduction:**

The public transport sector plays a crucial role in society, offering essential services and providing employment to a significant number of drivers. Despite the importance of this sector, it is essential to recognize that drivers are exposed to various occupational risks inherent to their daily work, which can have serious implications for their health. This study aims to characterize and analyse Work-Related Musculoskeletal Disorders (WMSD) and psychosocial risks in a public transport company.

**Methods:**

In the initial phase of the study, a questionnaire was administered to assess musculoskeletal symptoms and psychosocial risks. In the second phase, an inertial motion capture system was used to evaluate the risk of developing WMSD.

**Results:**

The results revealed a significant and concerning prevalence of burnout, with over 60% of workers reporting high or severe levels across all dimensions (i.e., personal, work-related, and client-related burnout). Depression, anxiety, and stress were within typical ranges, though a relevant percentage of participants exhibited severe and extremely severe levels of depression (7.2%), anxiety (12.2%), and stress (8%). Musculoskeletal discomfort was highly prevalent, particularly in the lower back (68.3%) and neck regions (57.2%), regarding pain over the last 12 months. Additionally, the risk of developing WMSDs was high across the various microtasks, which were analyzed across different bus lines and routes, with Rapid Upper Limb Assessment (RULA) scores ranging from 4 (Medium Risk) to 7 (Very High Risk).

**Discussion:**

Based on the results, varying bus types and routes is recommended. Programs should enhance wellbeing, and studies should assess interventions on health, stress, and occupational risks focused on enhancing worker wellbeing should be implemented, and future studies should assess the impact of interventions targeting health, stress, and occupational risks.

## 1 Introduction

The public transportation sector provides an essential service to the population and is responsible for ensuring employment for a significant number of drivers (1, 2). In the European Union, public bus transportation is widely used, reflecting its importance as the third most used mode of transportation (8.1%) in 2019, following air transport (9.7%) and cars (71.6%) (3). In 2021, Portugal had 118,322 professionals employed as heavy vehicle and bus drivers (4). Furthermore, according to Transport and Communications Statistics (*Estat*í*sticas dos Transportes e Comunicações*) (5), road passenger transport was the most prominent mode of transport in 2023, experiencing a 10.1% increase compared to 2022 and reaching a total of 547.7 million passengers (5).

According to Eurostat (6), the transport sector has the third-highest prevalence of work-related health problems in the European Community. Work-Related Musculoskeletal Disorders (WMSD) are one of these problems. In fact, they are one of the main occupational concerns across different professions. Data shows that approximately 10% of the general working population report health problems related to work (6). Of these, 60% referred to WMSD, which are the most common problem in Europe (6). Several studies emphasize the lower back as the area with the highest prevalence of pain and WMSD among drivers (2, 7–12). Other frequently reported areas include the neck (10–13) knees (8, 10), upper back (8, 11), and shoulders (11). In 2015, WMSD posed a significant challenge for workers in the transport sector within the European Community. Lower back pain affected 46% of workers, upper limb issues were reported by 37%, and lower limb problems by 26% of workers. These figures underscore the importance of Occupational Safety and Health (OSH) policies along with practices aimed at preventing and addressing these conditions to ensure the health and wellbeing of workers in this sector (14).

Workers in the transport sector are exposed to several risk factors that can contribute to the development of WMSD. These factors can be categorized into physical and biomechanical, organizational and psychosocial, and individual, as shown in Table 1. Irregular shifts, combined with frequent inadequate rest periods (15, 16), are frequently pointed in the literature as relevant risk factors in this profession. Additionally, long hours of sitting, performing complex maneuvers that require awkward postures, and exposure to whole-body vibration (WBV), all contribute to muscle fatigue, pain, and discomfort, particularly in the lower back, neck, shoulders, and knees (2, 7). These conditions increase the likelihood of developing WMSD, which are among the most frequently reported occupational health issues in Europe (8–10).

**Table 1:** Risk factors for the development of WMSD associated with the profession of driver.

**Category**	**Risk factor**	**Citation**
Physical and biomechanical	Repetitive movements of the upper limbs	(7)
	Prolonged periods of sitting	(2, 7, 51)
	Maintenance of incorrect, static, forced, or extreme postures	
	Whole-body vibration (WBV)	(2, 7)
	Spaces with inadequate dimensions	(2, 7, 9)
Psychosocial	High psychological and physical demands	(19, 55)
	Excessive working hours	(9, 10, 19, 51, 55)
	Fast-paced work environment	(17)
	Insufficient breaks for rest or opportunities to change posture	(7)
	Poor relationships with passengers	(20, 21)
	Work-related stress	(45, 55)
Individual	Years of experience as a driver	(2, 9, 10, 51)
	Medical history	(17)
	Physical fitness	
	Habits and lifestyle	
	Age	(9, 51)
	Body mass index (BMI)	

Additionally, beyond the physical demands of their tasks, drivers are also exposed to organizational and psychosocial stressors, such as night shifts, high work demands, and job insecurity, which can further exacerbate their health risks (17). However, these factors are also related to psychosocial risks. Furthermore, factors such as intimidation, harassment, and discrimination are prevalent in the occupational, context (17) particularly within the transport sector, which, in 2021, reported the highest incidence of discrimination cases and one of the highest rates of verbal abuse (18). These factors are strongly associated with the development of sleep disturbances, fatigue (19, 20), as well as psychological outcomes such as depression, anxiety, occupational stress (19), and burnout (21).

Considering the above, psychosocial factors contribute both to the risk of WMSD and to psychosocial risks. However, the relationship between psychosocial factors and WMSD is not necessarily negative. For example, self-confidence can potentially reduce stress impacts, with organizational, emotional, and social factors playing a significant role in these challenges (22–25). Given the well-established association between physical and mental health, this study also seeks to determine whether musculoskeletal symptoms correlate with psychosocial systems (57).

Individual risk factors are also relevant risk factors for WMSD and psychological disorders. This study will focus on age, body mass index (BMI), and years of professional experience. Previous studies indicate that age is a significant factor in WMSD (22) and in psychosocial symptoms prevalence, justifying investigation into whether older drivers experience higher health disorders (26, 27). Similarly, high BMI is associated with increased mechanical stress on joints, which may exacerbate the development of WMSD (22, 26, 27).

BMI has also been linked to poor mental health, including symptoms like somatization and impulsivity, contributing to a complex cycle of mental health issues. (28). Conditions such as depression and anxiety can lead to disordered eating (e.g., emotional eating), which in turn contributes to BMI increase, creating a self-reinforcing cycle. (28). Moreover, a study by Eik-Nes et al. (28), found an association between anxiety and higher BMI among the older adults, likely due to body's stress response system. Psychosocial stress can influence disordered eating behaviors, like binge eating, which serve as coping mechanisms. This can lead to challenges in managing weight over the long term. This justifies the analysis of BMI role in WMSD and psychosocial symptoms (28, 29).

Professional experience has also been related with an increase of musculoskeletal symptoms (22) and psychosocial symptoms (23–25). Longer work experience can lead to higher exposure to occupational risk factors, increasing the risk of WMSD, stress, burnout, and emotional burdens (22–25).

Psychosocial risks can also be related to each other. Cates (30) demonstrated that symptoms of depression, anxiety, and stress are strongly associated with higher levels of burnout, with these mental health issues playing a significant role in the emotional and physical exhaustion experienced. Thus, it becomes interesting to study this relationship as well.

Therefore, the aim of this study is to characterize and analyse WMSD and psychosocial risks among bus drivers in a public transport company. Additionally, the study seeks to explore the influence of individual risk factors, such as age, BMI, and years of service as a driver on musculoskeletal and psychosocial symptoms. Accordingly, the following hypotheses were formulated:

H1: The prevalence of musculoskeletal and psychosocial symptoms increases with drivers' age.

H2: Drivers with a higher BMI have a higher prevalence of musculoskeletal and psychosocial symptoms.

H3: Drivers with more years of service exhibit a higher prevalence of musculoskeletal and psychosocial symptoms.

H4: There is a positive correlation between the prevalence of musculoskeletal and psychosocial symptoms.

Given that two scales were employed to assess psychosocial risks, the following hypothesis was also tested:

H5: Symptoms of depression, anxiety, and stress are correlated with higher levels of burnout among drivers.

## 2 Materials and methods

### 2.1 Study design

This observational study was carried out in collaboration with a municipal public transport company.

In the first phase of the study, self-reported musculoskeletal pain symptoms among bus drivers were characterized, and the associated psychosocial risks were examined using an anonymous questionnaire. In the second phase, WBV exposure and the risk of developing WMSD were assessed using an inertial motion capture system. The tools and methodologies applied in each phase are summarized in Table 2.

**Table 2:** Description of the study phases.

**Study phases**	**Activities**
**1**^st^ **Phase**	
Preparation	Development of tools to support data collection, namely: - Development of the questionnaire for sociodemographic characterization and activity; - Selection of scales and tools to be used to characterize self-reported musculoskeletal pain symptomatology and psychosocial risks; - Selection of assessment methods for the risk of MSDs (Musculoskeletal Disorders) to be utilized.
Characterization	Application of the questionnaire. Assessment of the dimensions of Burnout through the Copenhagen Burnout Inventory; and of Depression, Anxiety, and Stress using the Depression, Anxiety, and Stress Scale (DASS-21); Characterization of musculoskeletal pain symptomatology using the Nordic Musculoskeletal Questionnaire.
Data analysis	Analysis of the data from the questionnaire results.
Results	Presentation of the results obtained internally.
**2**^nd^ **Phase**	
Preparation	Selection of assessment methods to be used—Xsens; Development of tools to support data collection, namely: Development of an Excel file to assist in data collection; Selection of the drivers to participate in the study. Selection of the routes and buses to be evaluated.
Characterization	Characterization of the risk of developing MSDs (Musculoskeletal Disorders) using Xsens.
Data analysis	Analysis of the data collected by Xsens.

The project received approval from the Ethics Committee of the School of Health at the Polytechnic Institute of Porto (CE0025E).

### 2.2 Sample

The study was conducted at a public transport bus company in northern Portugal that employs a total of 932 drivers, with 545 based at Bus Depot A and 387 at Bus Depot B. In the first phase of this study, 151 anonymous questionnaires were collected, comprising 61 (40.4%) from Bus Depot B and 90 (59.6%) from Bus Depot A. This sample represents approximately 16% of the company's total workforce with a margin of error of 6%. However, after analyzing the responses, only 139 questionnaires were considered valid for analysis. Questionnaires with incomplete responses were excluded.

Regarding the second phase of the study, the drivers, buses, and routes to be evaluated were selected by the company in coordination with the research team, among the ones that voluntarily accepted to be part of the study. The selection of drivers considered their anthropometric characteristics, and the type of bus they drove (standard and articulated, as well as newer and older models). The study included four male drivers representing extremes of age and height: two younger and two older, as well as two short and two tall individuals. Table 3 presents these characteristics. The buses used in the study were registered in 2007 and 2018, and all were equipped with adjustable seats and suspension systems.

**Table 3:** Characteristics of study participants (Phase II).

**Participant**	**Height (cm)**	**Length of service**	**Weight (category)**	**Age (Years)**
1	175	1.5	Obesity	27
2	170	5	Healthy Weight	36
3	168	25	Healthy Weight	46
4	182	3	Overweight	48

The selection of routes took into account the complaints reported by drivers regarding difficulties in driving due to pavement conditions. These routes displayed variations in pavement type (bitumen and cobblestones) and maintenance status, with certain sections of the circuit being considerably degraded. Regarding duration, two routes were shorter [mean (x) = 80 min; standard deviation (sd) = 9.51 min], while the other two had a longer duration [mean (x) = 103 min; standard deviation (sd) = 3 min].

To protect the privacy of the participants in the study, all workers were assigned identification codes comprising a letter followed by a digit (e.g., P1).

### 2.3 Instruments

#### 2.3.1 Questionnaire for sociodemographic and activity characterization

A questionnaire (available as Supplementary material) was developed and administered to collect sociodemographic data and information about the nature of the activities of the workers involved in the study. The questionnaire consisted of two distinct sections: (1) sociodemographic characterization, which compiled individual information from the professionals (such as age, sex, weight, height, and level of physical activity); and (2) activity characterization, which gathered information regarding the length of professional service and other characteristics related to the participants' work activities.

#### 2.3.2 Copenhagen burnout inventory

The Copenhagen Burnout Inventory (CBI) was developed by Kristensen et al. (31) and adapted into Portuguese by Fonte (32). This questionnaire is designed to psychometrically assess the levels of physical and psychological fatigue, as well as burnout exhaustion (31, 32).

The questionnaire comprised 19 items distributed across the three dimensions of burnout: (1) personal burnout, which evaluates the level of physical and psychological fatigue and individual exhaustion (six questions); (2) work-related burnout, which focuses on the degree of physical and psychological fatigue and exhaustion resulting from work (seven questions); and (3) client-related burnout, which estimates the physical and psychological fatigue and exhaustion caused by interactions with clients/passengers (six items). Participants' responses were rated on a Likert scale from 1 to 5. The response options included two formats: (1) 1 = always; 2 = often; 3 = sometimes; 4 = seldom; and 5 = never/almost never; and (2) 1 = To a very high degree; 2 = To a high degree; 3 = Somewhat; 4 = To a low degree; and 5 = To a very low degree (31).

In this study, Cronbach's alpha reliability coefficients for the CBI subscales were determined and found to be satisfactory (α personal = 0.907; α work-related = 0.851; and α client-related = 0.881).

#### 2.3.3 Depression, anxiety, and stress scale (DASS-21)

The Depression, Anxiety, and Stress Scale—DASS-21 was developed by Lovibond and Lovibond (33) and adapted into Portuguese by Apóstolo et al. (34). This questionnaire is designed to assess symptoms of depression, anxiety, and stress and is thus divided into three subscales: (1) depression, which evaluates the degree of hopelessness, lack of interest, low self-esteem, and sadness; (2) anxiety, which measures the level of nervousness, tension, fear, and feelings of panic; and (3) stress, which assesses the perception of overload, irritability, impatience, and difficulty relaxing (33, 34). Each subscale consists of 7 items, with participants rating each item on a Likert scale from 0 to 3 (0 = Did not apply to me at all; 1 = Applied to me to some degree, or some of the time; 2 = Applied to me to a considerable degree, or a good part of time; and 3 = Applied to me very much, or most of the time) according to the frequency with which the symptoms are experienced (33).

In this study, the Cronbach's alpha reliability coefficients for each subscale were also found to be good (depression α = 0.906; anxiety α = 0.900; and stress α = 0.913).

#### 2.3.4 Nordic musculoskeletal questionnaire

The Nordic Musculoskeletal Questionnaire, developed by Kuorinka et al. (35), was applied in this study in its validated version for the Portuguese language by Mesquita et al. (36). This questionnaire is a standardized tool developed to characterize the intensity and frequency of musculoskeletal symptoms among workers across nine body regions: neck, shoulders, elbows, wrists/hands, upper back, lower back, hips/thighs, knees, and ankles/feet (35, 36).

The questionnaire consists of 36 items that address all the previously mentioned body segments. Twenty-seven questions are dichotomous (yes or no) and organized into three categories: (1) in the last 12 months, have you experienced any problems (such as pain, discomfort, or numbness)?; (2) in the last 7 days, have you had any problems?; and (3) in the last 12 months, have you encountered difficulties in performing your normal activities (work, household tasks, or hobbies) due to musculoskeletal problems?. The remaining nine questions provide a pain Likert scale from 0 to 10 (0 = no pain and 10 = maximum pain), allowing participants to indicate the level of pain experienced by selecting a value on this scale (35, 36).

To facilitate the understanding of the nine body regions, the questionnaire includes an illustrative figure representing these anatomical areas (35).

### 2.4 Assessment of the risk of developing WMSD

To assess the risk of developing WMSD, an inertial motion capture system was used. Kinematic analysis was conducted using the Xsens MVN full-body motion capture system (Xsens Technologies, Enschede, Netherlands), consisting of 17 inertial measurement unit sensors that monitor movement in real time during tasks. This system provides data on 3D joint angles, center of mass, as well as temporal parameters, such as segment position, facilitating gait analysis.

For each participant, anthropometric data were collected, including measures such as height and shoe length. The data points collected were subsequently used to construct the MVN human model utilized with Xsens.

**Figure 1:**
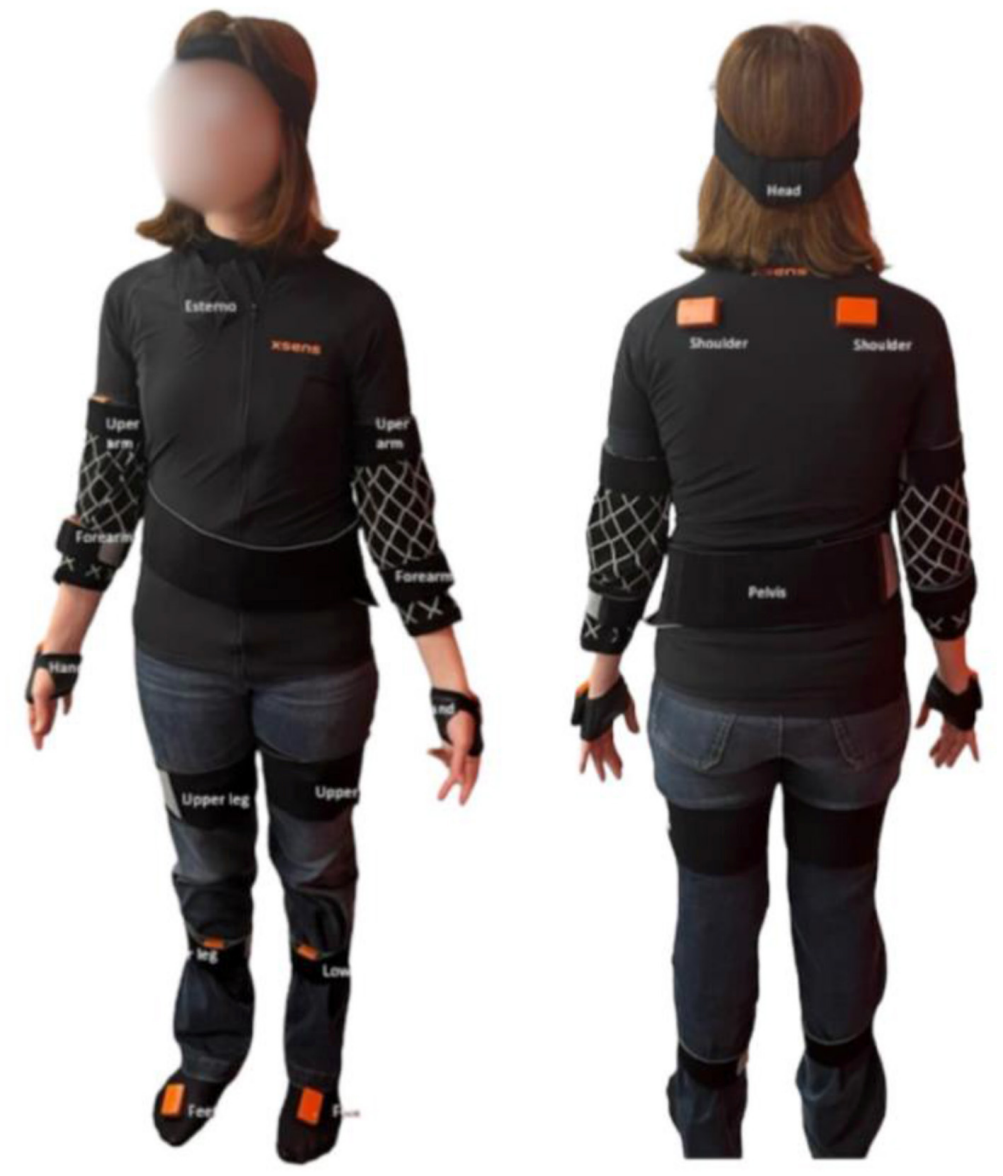
Placement of the straps and inertial sensors of the Xsens MVN.

After placing the sensors on the participants' body landmarks, calibration procedures were meticulously executed, strictly following the previously established protocol developed considering previous studies (37, 38) and the guidelines outlined in the Xsens Manual (39). In Figure 1, the placement of the straps and inertial sensors along the body is shown. Subsequently, each participant began the bus driving process while their postures and movements were monitored in real time. The obtained data were wirelessly sent to a computer equipped with software capable of observing, recording, and analyzing the movements. The data was processed using Xsens MVN software version 2021 (Xsens Technologies, Enschede, Netherlands).

This analysis involved the use of graphical representations, displaying joint angles, as well as evaluating movement speed and duration. Subsequently, values related to the Rapid Upper Limb Assessment (RULA) were determined, which focuses on the upper limbs (40).

The RULA action levels associate scores with risk levels to assess the urgency of adjusting work practices. A score of 1–2 indicates an acceptable posture, while a score of 3–4 suggests further investigation may be needed. Scores of 5–6 highlight the need for research and changes soon, and a score of 7 signals the need for immediate action due to a high risk of injury (40). These levels help identify when corrective measures are necessary to prevent musculoskeletal disorders. The final RULA scores were subsequently presented in tabular form.

### 2.5 Data analysis

The data collected with Xsens were exported to an Excel file for each microtask, and part of this data was then stored in a separate spreadsheet, where a semi-automated risk assessment is performed by integrating movement data from the inertial measurement units and manually inputting “muscle” and “force” values for RULA.

The Statistical Package for Social Sciences (SPSS) software version English 29.0.1.0 was used as a tool for data treatment and analysis. After checking and validating the data for errors, they were subsequently analyzed and processed. A descriptive analysis of the data was initially performed in SPSS to determine central tendency parameters (mean, standard deviation—sd, maximum, and minimum) and absolute and relative frequencies.

Inferential tests were also applied. The normality of the results was verified through the Kolmogorov-Smirnov test. Since the statistical distribution of all parameters was not normal, except for the variable “client-related burnout,” correlation coefficients were calculated using Spearman correlations, a non-parametric test.

The Spearman correlation coefficient, used to evaluate the relationship between two variables to determine the presence of a linear association, was applied to analyse the correlations between the following variables: age, length of service, body mass index, frequency of physical activity, musculoskeletal symptoms, symptoms of depression, anxiety, stress, burnout, and symptoms of WMSD. The established significance level was *p* < 0.05.

## 3 Results

### 3.1 Sociodemographic characterization

The sociodemographic characterization of the sample included in the first phase of this study is detailed in Table 4. The age group of 40 to 59 years was the most representative (> 70%). The average age was 45.99 ± 8.90 years, ranging from a minimum of 25 years to a maximum of 66 years. Regarding the length of employment in the company, the average number of years worked as drivers was 14.78 ± 11.36 years. However, 43.2% of the sample had worked for the company for between 1 and 4 years. In terms of nutritional status, Table 5 shows that a large portion of the participants (41.8%) were overweight, and 20.9% were classified as Obesity Class I.

**Table 4:** Sociodemographic characterization of the participants in the study (Phase I).

**Variables**	**N**	**Average**	**Sd**	**Maximum**	**Minimum**
Years (Age)	139	45.99	8.90	66	25
Weight (kg)	136	82.31	14.20	127	54
Height (cm)	137	174.00	7.70	197	156
Length of service (Years)	137	14.78	11.36	40	0.18

**Table 5:** Nutritional status of the participants of the study (Phase I) [Adapted from Weir and Jan (56)].

**BMI (kg/m^2^)**	**Nutritional status**	**N**	**(%)**
< 18.5	Underweight	0	0
18.5–24.9	Normal weight	46	34.3
25.0–29.9	Overweight	56	41.8
30.0–34.9	Class I Obesity	28	20.9
35.0−39.9	Class II Obesity	3	2.2
≥40	Class III Obesity	1	0.7

Regarding physical activity, 42 drivers (31.3%) indicated they never engage in physical exercise, while almost half of the sample (*n* = 61) reported exercising once or twice a week. Regarding hand dominance, most of the sample (86.5%) indicated being right-handed, while 9% identified as ambidextrous and 4.5% as left-handed.

### 3.2 Descriptive analysis of psychosocial and musculoskeletal symptom data

Regarding the CBI results, these are described by dimension in Table 6. The data show that the majority of surveyed workers reported high or severe levels of burnout across all three dimensions.

**Table 6:** Burnout levels assessed through the evaluation (Phase I).

**CBI**	**Personal (%)**	**Work-Related (%)**	**Client-Related (%)**
No/Low	3.6	0.7	2.2
Moderate	33.8	21.2	16.8
High	41.7	46.7	39.4
Severe	20.9	31.4	41.6
N	139	137	137

In relation to DASS-21, the results presented in Table 7 show that the majority of participants (75.5%, 71.2%, and 78.3% respectively) displayed normal levels of depression, anxiety, and stress. However, there is a notable presence of severe (4.3%) and extremely severe (7.9%) levels of anxiety. Additionally, 10.1% of subjects exhibited moderate levels of anxiety. This indicates that anxiety is a significant issue for a considerable portion of the workers studied. In addition, it is noteworthy that 7.2% of respondents showed severe or extremely severe levels of depression and 8% of stress.

**Table 7:** DASS-21 levels obtained from the assessment conducted (Phase I).

**DASS-21**	**Depression (%)**	**Anxiety (%)**	**Stress (%)**
Normal	75.5	71.2	78.3
Mild	12.2	6.5	5.8
Moderate	5.0	10.1	8.0
Severe	2.2	4.3	5.8
Extremely severe	5.0	7.9	2.2
N	139	139	138

### 3.3 Musculoskeletal symptom prevalence analysis

**Figure 2:**
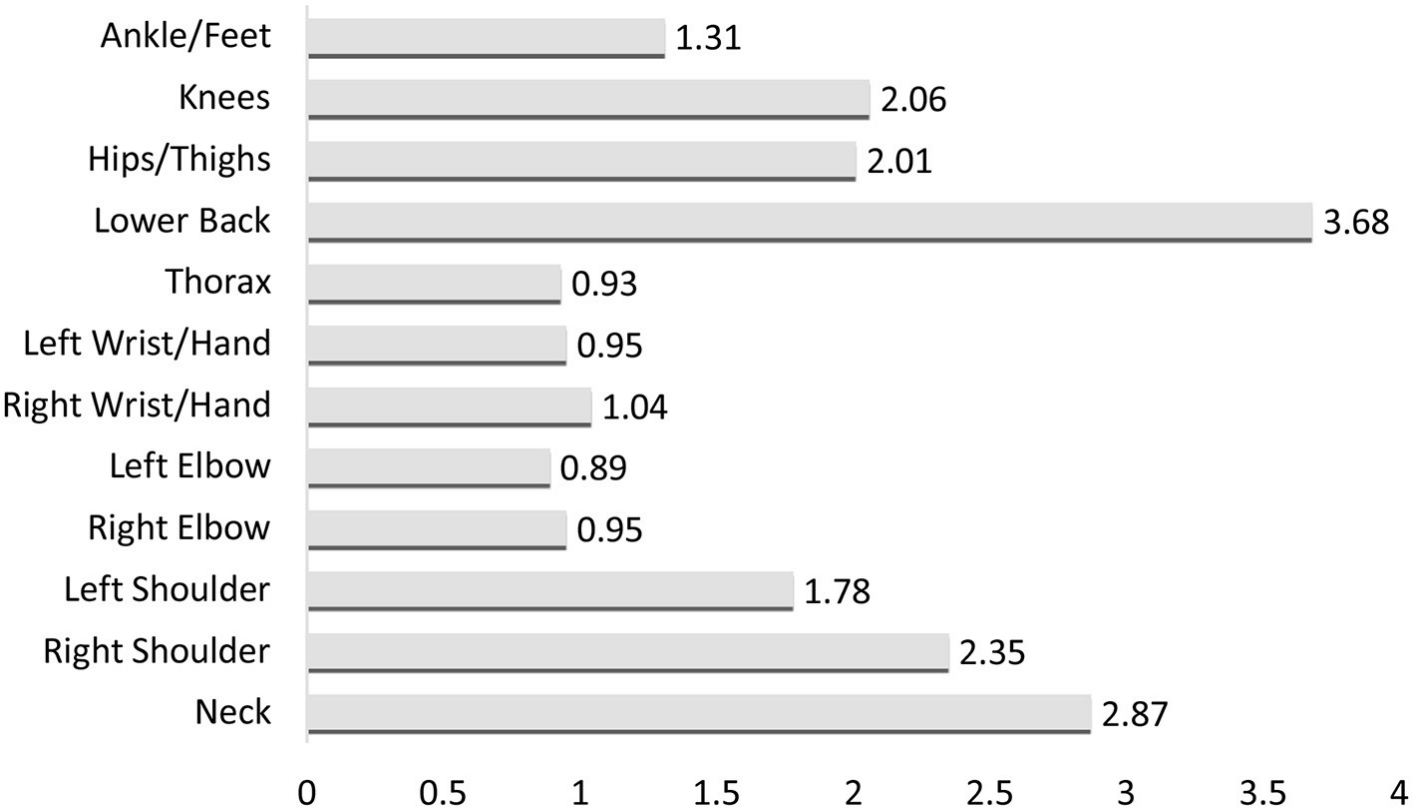
Average intensity of musculoskeletal pain.

The prevalence of musculoskeletal symptoms was also analyzed. The data presented in Table 8 and Figure 2 show that the lower back was the body region with the highest prevalence and intensity of musculoskeletal pain among the bus drivers studied. Over the past year, 68.3% of participants reported pain in the lower back, while 37.4% experienced this pain in the past week. The average intensity of pain in this region was the highest [mean (x) = 3.68], standing out as the area with the most intense pain. This pain is not only prevalent but also interfered with the daily activities of 40.5% of the sample.

**Table 8:** Prevalence and conditioning for WMSD obtained through the assessment conducted (Phase I).

**Variable**	**Pain over the last 12 months**		**Pain over the last 7 days**		**Limitation over the last 12 months**	
	**Yes (N)**	**(%)**	**Yes (N)**	**(%)**	**Yes (N)**	**(%)**
Neck	79	57.2	27	20.8	33	24.8
Right shoulder	55	39.9	21	16.2	28	20.9
Left shoulder	38	27.3	19	14.6	20	14.9
Right elbow	19	13.7	6	4.9	10	7.6
Left elbow	18	12.9	11	8.9	12	9.1
Right wrist/hand	28	20.1	11	8.8	17	12.9
Left wrist/hand	22	15.8	10	8.0	15	11.4
Thorax	23	16.5	14	11.2	13	9.8
Lower back	95	68.3	46	37.4	53	40.5
Hips/Thighs	52	37.7	26	20.8	30	22.7
Knees	51	37.0	25	20.2	30	22.9
Ankles/Feet	45	32.4	20	16.0	24	18.2

Regarding neck pain, there was a prevalence of 57.2% in the last 12 months, but this rate decreased to 20.8% in the last 7 days. Other affected areas included the right shoulder, hips/thighs, and knees, all with pain prevalence rates around 40% in the past year and 20% in the past week. Nearly 25% of participants reported that pain in the neck, hips/thighs, and knees interfered with their daily activities over the past year.

### 3.4 Analysis of WMSD development risk

The driving task was evaluated, being divided into 190 microtasks, and the results are summarized in Table 9. Examples of the microtask include turning right on a cobblestone surface, making a left turn on a tarmac surface with potholes, entering a roundabout and exiting at the first exit on a tarmac surface, among others. To determine the final RULA score, the average of the RULA values for each microtask was calculated for each route.

**Table 9:** Ergonomic risk assessment (Phase II).

**Participant**	**Bus**	**Route**	**RULA score**		**Risk level**	**Action to be taken**
			**Left side**	**Right side**		
1	Standard- 5^th^ Series MAN Gas (2018)	R1	Between 4 and 6 (predominantly 5)	Between 4 and 6 (predominantly 5)	Medium	Further investigation, change soon.
2	Articulated MAN Gas (2007)	R2	7	7	Very High	Investigate and implement changes.
3	Standard- 4^th^ Series MAN Gas (2007)	R3	Between 6 and 7 (predominantly 7)	Between 6 and 7 (predominantly 7)	Very High	Further investigation, change soon.
4	Standard- 4^th^ Series MAN Gas (2007)	R4	Between 6 and 7 (predominantly 7)	Between 6 and 7 (predominantly 7)	Very High	Investigate and implement changes.

The data obtained show final RULA scores ranging between 4 and 7. It was observed that the most recent bus had lower RULA scores (4), while the highest score (7) occurred when driving on a pothole-covered road.

Based on the analysis of movement data collected from the sensors, we determined the RULA scores, which revealed the following risk levels under the specified conditions:

- Bus Route 1, operated by a standard 5 ft Series MAN Gas bus manufactured in 2018, had a RULA score between 4 and 6 (predominantly 5), classified as Medium Risk Level.
- Bus Route 2, operated by an articulated MAN Gas bus manufactured in 2007, had a RULA score of 7, classified as Very High Risk.
- Bus Route 3, operated by a standard 4th Series MAN Gas bus manufactured in 2007, had a RULA score between 6 and 7 (predominantly 7), classified as Very High Risk.
- Bus Route 4, operated by a standard 4th Series MAN Gas bus manufactured in 2007, had a RULA score between 6 and 7 (predominantly 7), classified as Very High Risk.

To verify whether the type of bus influences the results, we conducted new assessments with the same drivers and on the same route, but swapping 2 standard buses between the routes. A total of 145 microtasks were analyzed and the conditions evaluated were:

- Bus Route 1, operated by a standard 4th Series MAN Gas bus manufactured in 2007, had a RULA score between 4 and 6 (predominantly 6), classified as Very High Risk.
- Bus Route 4, operated by a standard 5ft Series MAN Gas bus manufactured in 2018, had a RULA score between 6 and 7 (predominantly 7), classified as Very High Risk.

The results of this second assessment, present in Table 10, showed that the use of an older bus on route 1 increased the Risk Level from Medium to Very High. On the other hand, the use of a newer bus on route 4 did not reduce the risk level, suggesting that the Very High Risk Level is more related to the conditions of the route than to the bus itself.

**Table 10:** Ergonomic Risk Assessment after Bus Change between Routes (Phase II).

**Participant**	**Bus**	**Route**	**RULA Score**		**Risk level**	**Action to be taken**
			**Left side**	**Right side**		
1	Standard- 4^th^ Series MAN Gas (2007)	R1	Between 4 and 6 (predominantly 6)	Between 4 and 6 (predominantly 5)	Very High	Investigate and implement changes.
4	Standard- 5^th^ Series MAN Gas 20 (2018)	R4	Between 6 and 7 (predominantly 7)	Between 6 and 7 (predominantly 7)	Very High	Investigate and implement changes.

### 3.5 Relationship between psychosocial symptoms and demographic and behavioral variables

The relationship between demographic and behavioral variables and burnout sub-dimensions, depression, anxiety, and stress were analyzed, with the results presented in Table 11.

**Table 11:** Correlation of psychosocial symptoms with demographic and behavioral variables.

**Variable**	**Age**	**BMI**	**Years of work as a driver at the company**	**Frequency of physical activity practice**
Personal burnout	−0.250^**^	−0.175^*^	−0.122	−0.144
Work-related burnout	−0.309^**^	−0.150	−0.176^*^	−0.125
Client-related burnout	−0.301^**^	−0.069	−0.200^*^	−0.167
Depression	−0.132	−0.074	−0.020	−0.161
Anxiety	−0.078	−0.038	−0.021	−0.072
Stress	−0.148	−0.150	0.021	−0.105

^**^The correlation is significant at the 0.01 level (2-tailed).

^*^The correlation is significant at the 0.05 level (2-tailed).

There was a negative and significant correlation between age and the different dimensions of burnout (*p* < 0.01). These results suggest that older drivers tend to report lower levels of burnout. BMI showed a negative and significant correlation with personal burnout (*r* = −0.175^*^, *p* = 0.043).

Years of service in the company exhibited negative and significant correlations with work-related burnout (*r* = −0.176^*^, *p* = 0.040) and client-related burnout (*r* = −0.200^*^, *p* = 0.019). This suggests that drivers with more years of experience tend to experience lower levels of work-related and client-related burnout. No relationship was found between the frequency of physical activity and burnout.

Regarding anxiety, stress, and depression, no significant correlations were observed with demographic and behavioral variables (*p* > 0.05).

### 3.6 Relationship between musculoskeletal symptoms and demographic, behavioral, and psychosocial symptoms

The relationship between musculoskeletal symptoms and demographic and behavioral variables was analyzed, with the results shown in Table 12. The data indicates only a significant positive correlation between years of service and pain in the hips/thighs (*r* = 0.174^*^, *p* = 0.047), suggesting that more years working as drivers, the higher the musculoskeletal pain in this region. However, for other analyses, no significant correlations were found.

**Table 12:** Correlation of musculoskeletal symptoms with demographic and behavioral variables (Phase I).

**Variable**	**Age**	**BMI**	**Years of work as a driver for the company**	**Frequency of physical activity practice**
Neck	0.037	0.077	0.066	−0.145
Right shoulder	0.070	0.041	0.094	0.050
Left shoulder	0.047	0.032	0.083	0.015
Right elbow	0.028	−0.054	−0.013	−0.104
Left elbow	0.097	−0.068	0.099	−0.160
Right wrist/hand	0.033	0.026	0.137	−0.046
Left wrist/hand	0.033	−0.004	0.098	−0.089
Thorax	−0.036	−0.035	0.025	−0.065
Lower back	0.035	0.026	0.082	0.040
Hips/thighs	0.125	0.047	0.174^*^	−0.106
Knees	0.108	0.091	0.167	−0.125
Ankles/feet	0.018	0.022	0.108	−0.075

^*^The correlation is significant at the 0.05 level (2-tailed).

The results presented in Table 13 show the correlations between psychosocial symptoms and musculoskeletal symptoms in various body regions. Significant positive correlations were observed between the different dimensions of burnout, stress, anxiety, and depression with various body parts, with some exceptions. The strongest correlations were noted between the lumbar region and depression (*r* = 0.331^*^, *p* < 0.001), anxiety (*r* = 0.413^*^, *p* < 0.001), stress (*r* = 0.372^*^, *p* < 0.001), and the dimensions of burnout, followed by the thoracic region. Other areas of particular relevance include the neck, hips/thighs, and right shoulder. This indicates a linear association between increased psychosocial symptoms and increased pain in various body parts, particularly in the lumbar and thoracic regions.

**Table 13:** Correlation of musculoskeletal symptoms with psychosocial symptoms (Phase I).

**Variable**	**Depression**	**Anxiety**	**Stress**	**Personal burnout**	**Work-related burnout**	**Client-related burnout**
Neck	0.206^*^	0.227^**^	0.214^*^	0.399^**^	0.336^**^	0.230^**^
Right shoulder	0.193^*^	0.231^**^	0.241^**^	0.322^**^	0.314^**^	0.273^**^
Left shoulder	0.224^*^	0.193^*^	0.255^**^	0.326^**^	0.265^**^	0.245^**^
Right elbow	0.185^*^	0.198^*^	0.071	0.144	0.221^*^	0.124
Left elbow	0.121	0.154	0.077	0.110	0.098	0.070
Right wrist/hand	0.269^**^	0.270^**^	0.220^*^	0.272^**^	0.262^**^	0.206^**^
Left wrist/hand	0.254^**^	0.284^**^	0.262^**^	0.248^**^	0.210^*^	0.179^*^
Thorax	0.323^**^	0.309^**^	0.292^**^	0.394^**^	0.356^**^	0.258^**^
Lower back	0.331^**^	0.413^**^	0.372^**^	0.433^**^	0.336^**^	0.336^**^
Hips/thighs	0.266^**^	0.283^**^	0.282^**^	0.314^**^	0.320^**^	0.293^**^
Knees	0.168	0.217^*^	0.102	0.187^*^	0.228^**^	0.150
Ankles/feet	0.277^**^	0.228^**^	0.289^**^	0.229^**^	0.268^**^	0.199^*^

^**^The correlation is significant at the 0.01 level (2-tailed).

^*^The correlation is significant at the 0.05 level (2-tailed).

### 3.7 Correlation of DASS-21 data with CBI

The data presented in Table 14 reveal that all burnout subscales were significantly correlated with depression, anxiety, and stress. All correlations are positive and strong, indicating that high levels of burnout are associated with increased mental health issues.

**Table 14:** Correlation of DASS-21 Data with CBI (Phase I).

**Variable**	**Personal burnout**	**Work-related burnout**	**Client-related burnout**
Depression	0.626^**^	0.566^**^	0.565^**^
Anxiety	0.566^**^	0.524^**^	0.432^**^
Stress	0.674^**^	0.602^**^	0.584^**^

^**^The correlation is significant at the 0.01 level (2-tailed).

## 4 Discussion of results

The findings of this study highlight a significant prevalence of burnout among workers, which is consistent with previous research focusing on professions characterized by high demands and challenging work conditions, such as those in the transportation sector (41). These results suggest that burnout is a concern for workers in high-pressure environments but also a widespread issue in fields where job demands are intense, working hours are long, and personal time is limited (42). The emotional toll of such work environments appears to be a key driver of burnout, with many workers reporting severe symptoms (42).

Chen and Hsu (42) further noted that factors such as workload, work pace, and work-family conflict have a linear impact on emotional exhaustion. High levels of burnout have also been linked to lower job satisfaction, decreased organizational commitment, and an increased intention to leave the job (42). These factors point to a complex interaction between personal and work-related challenges that can significantly impact drivers' mental health. The presence of burnout in this population raises important concerns, especially given its association with decreased job satisfaction and the intention to leave the profession, as noted in other studies (42). The findings suggest that addressing these mental health issues through targeted interventions could improve drivers' wellbeing, reduce turnover, and enhance overall job performance.

Interestingly, our study also identified a notable presence of severe and extremely severe levels of anxiety, which were particularly noteworthy when compared to previous studies such as Apurva et al. (43). While Apurva et al. (43) primarily reported mild to moderate anxiety levels among female drivers, our study suggests that anxiety plays a more prominent role in affecting workers' mental health. This discrepancy may be attributed to differences in sample characteristics, as Apurva et al. (43) focused solely on female drivers, whereas our study predominantly examined a male sample. Gender differences could influence the manifestation of anxiety, potentially affecting how these emotions are experienced and expressed. In addition to gender, these differences may also be shaped by contextual and organizational factors, suggesting that the way each driver copes with the challenges of the profession could be influenced by multiple variables beyond personal characteristics (44).

Moreover, the results of this study align with previous studies that highlighted mental health challenges in professions involving high job demands and work stress (30, 45, 46). Other studies, such as Apurva et al. (43), reported moderate stress levels among bus drivers. However, this study was conducted with female drivers and our sample is dominated by males, which could influence the comparison of stress levels between the two groups. Useche et al. (45) reported that these stress levels are linked to insufficient time spent with family and lack of rest, both of which contribute to emotional strain and an imbalance between work and personal life. However, our results suggest that anxiety and burnout may play a more prominent role in drivers' mental health than stress. Specifically, the majority of our study participants displayed normal levels of stress, while a smaller portion exhibited severe and extremely severe levels of stress. This is noteworthy as it indicates that while stress levels were generally manageable, anxiety and burnout were more prevalent, requiring further attention.

In line with these findings, other studies, such as Batool et al. (41), have highlighted the influence of work conditions on drivers' mental health, particularly in relation to stress and burnout. Batool et al. (41), emphasized the negative impact of poor sleep quality and inadequate working conditions on drivers' wellbeing, and although sleep quality is a relevant variable, it was not considered in the present study. This study supports that notion by identifying dissatisfaction with bus ergonomics as a significant contributor to burnout levels. Previous research has suggested that factors such as old buses, exposure to vibrations, cabin noise, and lack of proper air circulation can exacerbate stress and contribute to burnout (41). Additionally, traffic congestion was found to significantly influence drivers' mental states, with studies indicating that in highly congested areas, drivers report increased feelings of depression, anger, and other negative emotions, which impair their perception, judgment, and driving actions, ultimately decreasing road safety (47, 48).

Regarding depression, a study by Dabholkar et al. (49) on bus drivers found 8% of severe depression, in contrast to our study, which found only 2.2%. Additionally, we identified higher levels of extremely severe depression (5%), whereas Dabholkar et al. (49) found no cases in this category. Contributing factors in their study included long hours with low pay, lack of incentives, disruptive shift rotations, monotonous work, and inadequate rest breaks, all of which negatively impacted both physical and mental wellbeing.

When analyzing the relationship between employees' age and psychosocial symptoms, burnout was the only factor significantly correlated with age. Data showed that older workers demonstrated lower burnout levels, rejecting hypothesis H1. Similarly, workers with more years of service as drivers experienced lower levels of work-related burnout, refuting hypothesis H3. These findings suggest that professional experience may contribute to greater resilience, as has been observed in another sector where workers with over 10 years of experience report lower levels of depression, anxiety, stress, and burnout. This trend is attributed to the development of greater resilience over time, allowing more experienced workers to manage adversities in ways that mitigate negative impacts on their mental health (30).

The association between higher BMI and the prevalence of musculoskeletal and psychosocial symptoms was found to be insignificant, leading to the rejection of H2 due to a lack of supporting evidence. This finding contrasts with studies that have linked higher BMI to an increased risk of physical and psychological health issues in various occupational settings. However, it is possible that other factors, such as physical activity levels or job-specific demands, play a more significant role in shaping these outcomes among bus drivers.

In addition to psychosocial factors, our study revealed a high prevalence of musculoskeletal symptoms among participants, particularly affecting the lumbar region and neck. These findings, align with previous research indicating that drivers are at a significant risk of developing work-related musculoskeletal disorders (WMSD), especially in these areas (2, 13). While our results corroborate studies that identified the lumbar region as the most affected body area among drivers, some discrepancies emerge when compared to other investigations. For instance, Kasemsan et al. (13) reported a higher prevalence of discomfort in the neck (81.9%) than in the lumbar region (80.7%), while Chen et al. (50) found greater discomfort in the neck (46.9%), right shoulder (40.0%), and lumbar region (37.2%). These variations may be attributed to differences in study populations, vehicle design, and driving conditions, underscoring the need for further research to better understand the specific risk factors contributing to these outcomes.

One of the key objectives of our study was to assess whether musculoskeletal symptoms increased with age, BMI, and professional experience. Contrary to some previous findings, our results did not establish a significant correlation between age or BMI and higher musculoskeletal pain levels. This is consistent with Hakim and Mohsen (9), who also found no significant association between BMI and lower back pain. However, other studies suggest contrasting trends; for example, Maduagwu et al. (7) and Laal et al. (8) reported a significant correlation between increased age and the prevalence of WMSD, particularly affecting the hips, hands, and thoracic region. Interestingly, Laal et al. (8) found an inverse relationship between BMI and musculoskeletal discomfort in the thoracic and lumbar regions, suggesting that body composition may play a complex role in WMSD development. Additionally, our study identified a correlation between increased years of service as a driver and heightened pain in the hips and thighs, which aligns with findings by Maduagwu et al. (7), who observed that workers with 1 to 5 years of employment exhibited a higher prevalence of WMSD.

Beyond individual characteristics, workplace conditions play a critical role in musculoskeletal health. Previous studies have identified insufficient breaks, working while injured, lifting or carrying heavy loads from passengers, and prolonged exposure to job demands as significant contributors to WMSD (7). While our study did not assess all possible risk factors, our findings support previous research by Ekechukwu et al. (51) and Abledu et al. (52), which highlighted work duration (hours/day), work frequency (days/week), job satisfaction, and work stress as key determinants of WMSD in drivers. Additionally, research by Hakim and Mohsen (9) found no significant relationship between lower back pain and the number of days worked per week, suggesting that other occupational variables, such as workload intensity and seating conditions, may have a greater impact on musculoskeletal health. Additional research suggests that awkward and unusual postures, fatigue, and high work pace also contribute to the development of musculoskeletal injuries (51). Furthermore, older workers with more than 10 years of experience and those who work more than 8 h/day have been found to have a higher prevalence of lower back pain (9). Stress and uncomfortable seating have also been linked to increased discomfort in the neck, shoulders, and lumbar region (50).

An important factor influencing the risk of developing WMSD is the type of bus used. Our analysis showed that older buses were associated with higher risks compared to newer models, which offer improved ergonomics, better postural support, reduced physical effort for drivers, and decreased exposure to vibration. Interestingly, our second assessment showed that using an older bus on route 1 led to an increase in the risk level, while the implementation of a newer bus on route 4 did not significantly decrease the risk level. This suggests that the higher risk on this route may be more closely linked to the specific conditions of the route rather than the bus's age or model. Further studies are necessary to assess the impact of the bus type on WMSD risk across different routes and with different drivers.

Our findings also highlight a strong association between musculoskeletal symptoms and psychosocial stressors. A positive correlation was found between musculoskeletal symptoms and depression, anxiety, stress, and all burnout dimensions, corroborating hypothesis H4. These correlations are particularly strong for the lumbar region. This supports previous research indicating a bidirectional relationship between mental health and musculoskeletal disorders, where psychological distress can exacerbate physical symptoms and vice versa. For instance, a study conducted among healthcare students found a significant association between burnout levels and WMSD, though it did not establish a direct link between lower back and neck pain with burnout (53). Similarly, Alsaadi (54) established correlations between anxiety, stress and musculoskeletal pain, with stress stemming from a lack of leisure time and anxiety resulting from the demands and workload required. Such issues can impair professional performance, induce muscle tension, and contribute to musculoskeletal pain (54).

Furthermore, our study found a significant positive correlation between results obtained from the DASS and the CBI, confirming hypothesis H5. Specifically, personal burnout dimension was statistically well correlated with stress and depression. These findings align with a study conducted by Cates et al. (30) on midwives, which also observed a similar correlation pattern. Similarly, Useche et al. (45) also found that emotional exhaustion was positively correlated with work tension/stress.

Ultimately, this study contributes to a deeper understanding of the occupational risks faced by bus drivers, particularly in relation to musculoskeletal disorders and psychosocial stressors. The findings reinforce the necessity of improving working conditions, promoting ergonomic interventions, and implementing preventive measures tailored to the specific demands of this profession. Notably, Pickard et al. (2) emphasized the lack of studies focusing on effective strategies to mitigate the risk of developing WMSD among bus drivers. Several studies suggest that targeted intervention programs, including occupational gymnastics and structured break schedules, could help reduce these risks (2, 8–10). In terms of psychosocial risk factors, Useche et al. (55) highlight the need for further research on workload and its impact on drivers' mental wellbeing. Addressing these multifaceted challenges through evidence-based interventions may enhance drivers' overall health, reduce turnover, and improve workplace sustainability.

## 5 Conclusions

The main objective of this study was to characterize the self-reported musculoskeletal pain symptoms and investigate the associated psychosocial risks among bus drivers. Additionally, the study aimed to assess the influence of other risk factors, such as age, BMI, and years of service as a driver, on musculoskeletal and psychosocial symptoms. The research successfully fulfilled its objective by exploring the risk factors to which drivers are exposed, highlighting the significance of psychosocial risks within this professional group.

The results revealed a high and severe prevalence of burnout, corroborating findings from previous studies in similar contexts. However, it was observed that levels of anxiety, depression, and stress among drivers were predominantly normal, contrasting with findings from other studies. The research also highlighted the high prevalence of musculoskeletal pain, especially in the lumbar region and neck, aligning with previous studies. Lastly, the microtask analysis revealed a high risk of developing WMSD among drivers. However, when analyzing the most recent bus, lower RULA values were observed, especially on regular pavements, indicating that more recent vehicles may contribute to the reduction of ergonomic risks. Nevertheless, in recent evaluations where we assessed the drivers on the same routes but exchanged buses between the routes, we found that using an older bus on route 1 increases the risk level. Similarly, using a newer bus on route 4 did not reduce the risk level, suggesting that the higher risk of the route is more related to its conditions than to the bus itself. Therefore, it is advisable to carry out a study to categorize the routes by risk level, allowing for adjustments in driver assignments accordingly. It is crucial to ensure that a driver is not solely allocated to a set of routes with a higher risk level, but rather that there is a rotation system in place to maintain balance. This approach helps mitigate the development of WMSDs and psychosocial risks.

The correlations between musculoskeletal pain and psychosocial symptoms were notably stronger, with musculoskeletal pain significantly linked to burnout, depression, anxiety, and stress. These findings underscore the need to improve working conditions and protect the health of bus drivers.

Considering the study's findings, it is recommended to develop intervention programs aimed at reducing musculoskeletal symptoms and workload for bus drivers. Such programs could include physical and psychological assessments to establish a baseline and measure potential changes over time. Incorporating structured physical activities, such as muscle stretching exercises, may help relieve tension and promote wellbeing. These activities could be integrated into the workday, with progress monitored through appropriate tracking methods. By addressing both physical and psychosocial risks, these programs would contribute to improving drivers' overall health and work conditions.

### 5.1 Study limitations

Despite its contributions, this study has certain limitations. Since these questionnaires are subjective, drivers may have underestimated or overestimated their symptoms or been reluctant to share certain experiences, potentially affecting the results. An additional limitation of this study could be selection bias, given that the employees who opted to complete the questionnaires might have been driven to participate due to experiencing more severe WMSD compared to those who chose not to take part.

The use of Xsens for data collection on specific routes with designated bus types may have limited the analysis, as variations between different buses on the same route were not considered. This could have provided a more comprehensive perspective on the effects of biomechanical risk factors on drivers' working conditions. Furthermore, the study focused only on psychosocial and biomechanical risks, overlooking other potential risks such as physical ones.

Lastly, multivariate analyses were not performed, and therefore the influence of potential confounders could not be adequately controlled.

### 5.2 Future research

Future studies should adopt a broader approach that addresses all major occupational risks faced by drivers, including physical and biological ones, which could provide a more comprehensive view of the occupational hazards drivers face. Data on WBV exposure should be collected across different routes and buses.

Forthcoming work should alternate the types of buses evaluated on a particular route, as well as switch the routes that the same bus type operates. Additionally, the sample size should be increased to obtain more robust and concrete data about the reality.

Considering the results obtained, it is essential to conduct longitudinal studies that develop and evaluate the effectiveness of ergonomic and psychological interventions to ascertain whether they contribute to reducing musculoskeletal symptoms, burnout, anxiety, depression, and stress among bus drivers. Furthermore, an assessment of the effectiveness of the occupational gymnastics plan should be conducted to determine its impact on drivers' physical and mental health.

## Data Availability

The datasets presented in this article are not readily available because in accordance with the requirements of the Ethics Committee, the collected databases must not be shared and should be deleted after publication. Any changes to this procedure must be submitted to the Ethics Committee for review. Requests to access the datasets should be directed to Tânia T. Silva, tania4teixeira@hotmail.com.

## References

[1] Montoro L Useche S Alonso F Cendales B. Work environment, stress, and driving anger: a structural equation model for predicting traffic sanctions of public transport drivers. Int J Environ Res Public Health. (2018) 15:497. 10.3390/ijerph15030497 29534530 PMC5877042

[2] Pickard O Burton P Yamada H Schram B Canetti E Orr R. Musculoskeletal disorders associated with occupational driving: a systematic review spanning 2006–2021. Int J Environ Res Public Health. (2022) 19:6837. 10.3390/ijerph19116837 35682420 PMC9180502

[3] EC. EU Transport in Figures—Statistical Pocketbook 2021. Luxembourg: Publications Office of the European Union (2021).

[4] INE. CENSOS 2021: Profissões e Escolaridade. (2023). Available online at: https://www.ine.pt/xportal/xmain?xpid=INE&xpgid=ine_destaques&DESTAQUESdest_boui=611923478&DESTAQUESmodo=2 (accessed April 5, 2024).

[5] INE. Estatísticas do Transportes e Comunicações–2023. Instituto Nacional de Estatística, I.P. (2024). Available online at: https://www.ine.pt/xportal/xmain?xpid=INE&xpgid=ine_publicacoes&PUBLICACOESpub_boui=439493357&PUBLICACOESmodo=2 (accessed February 13, 2025).

[6] Eurostat. Self-reported work-related health problems and risk factors - key statistics. (2021). Available online at: https://ec.europa.eu/eurostat/statistics-explained/index.php?title=Self-reported_work-related_health_problems_and_risk_factors_-_key_statistics#Most_affected_sectors_of_economy_and_groups_of_occupations (accessed April 5, 2024).

[7] Maduagwu S Galadima N Umeonwuka C Ishaku C Akanbi O Jaiyeola O. Work-related musculoskeletal disorders among occupational drivers in Mubi, Nigeria. Int J Occup Saf Ergon. (2022) 28:572–80. 10.1080/10803548.2020.1834233 33034261

[8] Laal F Madvari R Balarak D Mohammadi M Dortaj E Khammar A. Relationship between musculoskeletal disorders and anthropometric indices among bus drivers in Zahedan city. Int J Occup Saf Ergon. (2018) 24:431–7. 10.1080/10803548.2017.1334335 28589778

[9] Hakim S Mohsen A. Work-related and ergonomic risk factors associated with low back pain among bus drivers. J Egypt Public Health Assoc. (2017) 92:195–201. 10.21608/epx.2017.16405 30341998

[10] Lalit. The prevalence of musculoskeletal disorders among bus drivers in tricity. Int J Physiother. (2015) 2:850–4. 10.15621/ijphy/2015/v2i5/78244

[11] Joseph L Standen M Paungmali A Kuisma R Sitilertpisan P Pirunsan U. Prevalence of musculoskeletal pain among professional drivers: a systematic review. J Occup Health. (2020) 62:e12150. 10.1002/1348-9585.12150 32810918 PMC7434558

[12] Yasobant S Chandran M Reddy E. Are bus drivers at an increased risk for developing musculoskeletal disorders? An ergonomic risk assessment study. J Ergon. (2015) S3:1–5. 10.4172/2165-7556.S3-011

[13] Kasemsan A Joseph L Paungmali A Sitilertpisan P Pirunsan U. Prevalence of musculoskeletal pain and associated disability among professional bus drivers: a cross-sectional study. Int Arch Occup Environ Health. (2021) 94:1263–70. 10.1007/s00420-021-01683-1 33856539 PMC8047600

[14] Kok J Vroonhof P Snijders J Roullis G Clarke M Peereboom K. Work-related musculoskeletal disorders: prevalence, costs and demographics in the EU. European Agency for Safety and Health at Work (2019). Available online at: https://data.europa.eu/doi/10.2802/66947 (accessed April 5, 2024).

[15] Santos J Lu J. Occupational safety conditions of bus drivers in Metro Manila, the Philippines. Int J Occup Saf Ergon. (2016) 22:508–13. 10.1080/10803548.2016.1151700 27093582

[16] Wei C Gerberich S Ryan A Alexander B Church T Manser M. Risk factors for unintentional occupational injury among urban transit bus drivers: a cohort longitudinal study. Ann Epidemiol. (2017) 27:763–70. 10.1016/j.annepidem.2017.09.011 29126665

[17] EU-OSHA. Lesões musculoesqueléticas. (2024). Available online at: https://osha.europa.eu/pt/themes/musculoskeletal-disorders (accessed April 5, 2024).

[18] Gaude L Cabrita J Eiffe F Gerstenberger B Ivaškaite-Tamošiune V Parent-Thirion A. Working Conditions in the Time of COVID-19: Implications for the Future. European Working Conditions Telephone Survey 2021 series. Publications Office of the European Union (2022). Available online at: https://www.eurofound.europa.eu/en/publications/2022/working-conditions-time-covid-19-implications-future (accessed April 5, 2024).

[19] Cendales B Gómez-Ortiz V Useche S Cedillo L Stephenson D Landsbergis P. Mental health outcomes among urban public transport workers: a systematic literature review. J Transp Health. (2024) 36:101804. 10.1016/j.jth.2024.101804

[20] Hu SR Chen SY. Effects of mixed traffic and elderly passengers on city bus drivers' work-related fatigue. Transp Res Part F Traffic Psychol Behav. (2019) 66:485–500. 10.1016/j.trf.2019.09.020

[21] Tu Z He J Zhou N Shen X. Driver-passenger communicative stress and psychological distress among Chinese bus drivers: the mediating effect of job burnout. BMC Public Health. (2021) 21:547. 10.1186/s12889-021-10618-x 33743660 PMC7980616

[22] Kim IJ. An ergonomic focus evaluation of work-related musculoskeletal disorders amongst operators in the UAE network control centres. Heliyon. (2023) 9:e21140. 10.1016/j.heliyon.2023.e21140 37916099 PMC10616411

[23] Makara-Studzińska M Wadja Z Lizińczyk S. Years of service, self-efficacy, stress and burnout among Polish firefighters. Int J Occup Med Environ Health. 33:283–97. 10.13075/ijomeh.1896.01483 32210420

[24] Bergman B Mackay D Smith D Pell J. Long-term mental health outcomes of military service: national linkage study of 57,000 veterans and 173,000 matched nonveterans. J Clin Psychiatry. (2016) 77:793–8. 10.4088/JCP.15m09837 27135139

[25] Laditka J Laditka S Arif A Adeyemi O. Psychological distress is more common in some occupations and increases with job tenure: a thirty-seven year panel study in the United States. BMC Psychol. (2023) 11:95. 10.1186/s40359-023-01119-0 37004123 PMC10064628

[26] Demissie B Bayih E Demmelash A. A systematic review of work-related musculoskeletal disorders and risk factors among computer users. Heliyon. (2024) 10:e25075. 10.1016/j.heliyon.2024.e25075 38318034 PMC10840111

[27] Bispo L Moreno C Silva G Albuquerque Silva J. Risk factors for work-related musculoskeletal disorders: a study in the inner regions of Alagoas and Bahia. Saf Sci. (2022) 153:105804. 10.1016/j.ssci.2022.10580435661042

[28] Eik-Nes T Tokatlian A Raman J Spirou D Kvaløy K. Depression, anxiety, and psychosocial stressors across BMI classes: a Norwegian population study—the HUNT study. Front Endocrinol. (2022) 13:886148. 10.3389/fendo.2022.886148 36034441 PMC9399822

[29] Tambelli R Cerniglia L Cimino S Ballarotto G Paciello M Lubrano C. An exploratory study on the influence of psychopathological risk and impulsivity on BMI and perceived quality of life in obese patients. Nutrients. (2017) 9:431. 10.3390/nu9050431 28445437 PMC5452161

[30] Cates E Ramlogan-Salanga C MacKenzie R Wilson-Mitchell K Darling E. A cross-sectional survey of the mental health of midwives in Ontario, Canada: burnout, depression, anxiety, stress, and associated factors. Women Birth. (2024) 37:101613. 10.1016/j.wombi.2024.101613 38615516

[31] Kristensen T Borritz M Villadsen E Christensen K. The copenhagen burnout inventory: a new tool for the assessment of burnout. Work Stress. (2005) 19:192–207. 10.1080/02678370500297720

[32] Fonte C. Adaptação e validação para português do questionário de Copenhagen Burnout Inventory (CBI) [Dissertação de mestrado em Gestão e Economia da Saúde]. Universidade de Coimbra (2011). Available online at: https://hdl.handle.net/10316/18118

[33] Lovibond S Lovibond P. Manual for the Depression Anxiety Stress Scales. Sydney: Psychology. (1995). Available online at: https://psycnet.apa.org/doi/10.1037/t01004-000 (accessed August 21, 2023).

[34] Apóstolo L Mendes A Azeredo Z. Adaptation to Portuguese of the depression, anxiety and stress scales (DASS). Rev Lat Am Enfermagem. (2006) 14:863–71. 10.1590/S0104-11692006000600006 17294019

[35] Kuorinka I Jonsson B Kilbom A Vinterberg H Biering-Sørensen F Anderson G. Standardised Nordic questionnaires for the analysis of musculoskeletal symptoms. Appl Ergon. (1987) 18:233–7. 10.1016/0003-6870(87)90010-X 15676628

[36] Mesquita C Ribeiro J Moreira P. Portuguese version of the standardized Nordic musculoskeletal questionnaire: cross cultural and reliability. J Public Health. (2010) 18:461–6. 10.1007/s10389-010-0331-0

[37] Colim A Faria C Cunha J Oliveira J Sousa N Rocha L. Physical ergonomic improvement and safe design of an assembly workstation through collaborative robotics. Safety. (2021) 7:14. 10.3390/safety7010014

[38] Silva TT Sousa C Colim A Rodrigues MA. Understanding musculoskeletal loadings among supermarket checkout counter cashiers: a biomechanical analysis. Safety. (2024) 10:21. 10.3390/safety10010021

[39] Schepers M Giuberti M Bellusci G. Xsens MVN: Consistent Tracking of Human Motion Using Inertial Sensing. Vol. 1. Xsens Technologies (2018). p. 1–8. Available online at: 10.13140/RG.2.2.22099.07205

[40] McAtamney L Corlett E. RULA: a survey method for the investigation of work-related upper limb disorders. Appl Ergon. (1993) 24:91–9. 10.1016/0003-6870(93)90080-S 15676903

[41] Batool Z Younis M Yasir A Rehman A Dilawar M Yasin M. Effects of safety pattern, cabin ergonomics, and sleep on work-related stress and burnout of city and transit bus drivers in Lahore, Pakistan. Ergonomics. (2021) 65:704–18. 10.1080/00140139.2021.1983029 34544328

[42] Chen C Hsu YC. Taking a closer look at bus driver emotional exhaustion and well-being: evidence from Taiwanese urban bus drivers. Saf Health Work. (2020) 11:353–60. 10.1016/j.shaw.2020.06.002 32995061 PMC7502616

[43] Apurva G Smita C Chandrakant B Khushboo T. Prevalence of work related musculoskeletal and psychological problems among female bus conductors in Karad. Int J Life Sci Pharma Res. (2020) 10:L21–28. 10.22376/ijpbs/lpr.2020.10.4.L21-28

[44] Chen MY Kao CL. Women on boards of directors and firm performance: the mediation of employment downsizing. Int J Hum Resour Manag. (2021) 33:2597–629. 10.1080/09585192.2020.1867617

[45] Useche S Alonso F Cendales B Autukeviciute R Serge A. Burnout, job strain and road accidents in the field of public transportation: the case of city bus drivers. J Environ Ad Occup Sci. (2017) 6:1. 10.5455/jeos.20170202074636

[46] Ali K Rahman KSG Nancy S Kumar S. Mental health among bus drivers and conductors: a cross-sectional study from Karaikal, South India. Cureus. (2023) 15:e43273. 10.7759/cureus.43273 37692676 PMC10492572

[47] Bitkina O Kim J Park J Park J Kim H. Identifying traffic context using driving stress: a longitudinal preliminary case study. Sensors. (2019) 19:2152. 10.3390/s19092152 31075920 PMC6539244

[48] Larue G Blackman R Freeman J. Frustration at congested railway level crossings: how long before extended closures result in risky behaviours? Appl Ergon. (2020) 82:102943. 10.1016/j.apergo.2019.102943 31476605

[49] Dabholkar A Khatib S Dabholkar T. Psychological problems faced by Navi Mumbai bus conductors. Int J Comm Med Public Health. (2015) 2:184–8. 10.5455/2394-6040.ijcmph20150523

[50] Chen YL Alexander H Hu YM. Self-reported musculoskeletal disorder symptoms among bus drivers in the Taipei metropolitan area. Int J Environ Res Public Health. (2022) 19:10596. 10.3390/ijerph191710596 36078314 PMC9518195

[51] Ekechukwu E Useh E Nna O Ekechukwu N Obi O Aguwa E. Ergonomic assessment of work-related musculoskeletal disorder and its determinants among commercial mini bus drivers and driver assistants (mini bus conductors) in Nigeria. PLoS ONE. (2021) 16:e0260211. 10.1371/journal.pone.0260211 34874951 PMC8651118

[52] Abledu J Offei E Abledu G. Predictors of work-related musculoskeletal disorders among commercial minibus drivers in Accra metropolis, Ghana. Adv Epidemiol. (2014) 5:384279. 10.1155/2014/384279

[53] Alqahtani N Abdulaziz A Hendi O Mahfouz M. Prevalence of burnout syndrome among students of health care colleges and its correlation to musculoskeletal disorders in Saudi Arabia. Int J Prev Med. (2020) 11:38. 10.4103/ijpvm.IJPVM_295_19 32363025 PMC7187547

[54] Alsaadi S. Musculoskeletal pain in undergraduate students is significantly associated with psychological distress and poor sleep quality. Int J Environ Res Public Health. (2022) 19:13929. 10.3390/ijerph192113929 36360807 PMC9658124

[55] Useche S Gómez V Cendales B Alonso F. Working conditions, job strain, and traffic safety among three groups of public transport drivers. Saf Health Work. (2018) 9:454–61. 10.1016/j.shaw.2018.01.003 30559995 PMC6284153

[56] Weir CB Jan A. BMI Classification Percentile and Cut-Off Points. Treasure Island (FL): StatPearls Publishing (2024). Available online at: https://www.ncbi.nlm.nih.gov/books/NBK541070/ (accessed June 26, 2023). 31082114

[57] Nascimento J Bispo L Silva J. Risk factors for work-related musculoskeletal disorders among workers in Brazil: a structural equation model approach. Int J Ind Ergon. (2024) 99:103551. 10.1016/j.ergon.2024.103551

