# Supplementary material for: Assessing work-related musculoskeletal disorders and psychosocial risks in bus drivers: insights from a municipal company case study in Portugal
Source: Front Public Health. 2025 Apr 30;13:1529023. doi: 10.3389/fpubh.2025.1529023 (PMC12075220; doi:10.3389/fpubh.2025.1529023)
Supplement: Supplementary file 1 [file Data_Sheet_1.pdf]

ErgoBus: Prevenção de LMERT em motoristas de autocarros

Questionário

Este questionário tem como objetivo caracterizar a sintomatologia de dor musculosquelética em motoristas de autocarros, bem como os riscos psicossociais. Todos os dados pessoais recolhidos no âmbito do presente questionário serão utilizados única e exclusivamente para os objetivos deste estudo, garantindo-se que o seu tratamento será realizadode forma anonimizada.

Grupo I – Caracterização sociodemográfica

1. Idade ____Anos **2.** Peso _____Kg **3.** Altura ______Cm

4. Considerando a habilidade da mão, é: ☐ Destro(a) ☐ Esquerdino(a) ☐ Ambidestro(a)

5. Pratica algum desporto ou atividade física?

☐ Nunca ☐ 1 a 2 vezes por semana ☐ 3 a 4 vezes por semana ☐ > 4 vezes por semana

Grupo II – Caracterização da atividade

1. Há quantos anos trabalha na empresa como motorista: _____anos

2. Qual o mapa de serviço (grupo de linhas) que costuma normalmente realizar? __________________

3. Qual(ais) o(s) tipo(s) de autocarro (s) que costuma conduzir?
____

Grupo III – Riscos psicossociais

Copenhagen Burnout inventory (Fonte, 2011; Kristensen et al., 2005)

O questionário seguinte destina-se à avaliação psicométrica das dimensões do *Burnout*. Não existem respostas melhores ou piores; a resposta correta é aquela que exprime com veracidade a sua própria experiência.

A cada uma das frases deve responder consoante a frequência com que tem esse sentimento selecionando a caixa adequada.

Item	Sempre	Frequentemente	Às vezes	Raramente	Nunca/quase nunca
1.Está cansado de trabalhar com clientes?*					
2.Com que frequência se sente cansado(a)?*					
3.Sente-se esgotado(a) no final de um dia de trabalho?					
4.Com que frequência se sente fisicamente exausto(a)?*					
5.Sente-se exausto(a) de manhã ao pensar em mais um dia de trabalho?					
6.Alguma vez se questiona quanto tempo conseguirá continuar a trabalhar com clientes?*					

ErgoBus: Prevenção de LMERT em motoristas de autocarros

7.Com que frequência se sente emocionalmente exausto(a)?					
8.Sente que cada hora de trabalho é cansativa para si?					
9.Com que frequência pensa: "Eu não aguento mais isto"?					
10.Com que frequência se sente fatigado(a)?					
11.Com que frequência se sente frágil e suscetível a ficar doente?					
12.Tem energia suficiente para a família e os amigos durante o tempo de lazer?					

Item	Muito	Bastante	Assim, assim	Pouco	Muito Pouco
13.O seu trabalho é emocionalmente desgastante?					
14.O seu trabalho deixa-o frustrado(a)?					
15.Acha difícil trabalhar com clientes?*					
16.Acha frustrante trabalhar com clientes?*					
17.Sente-se esgotado por causa do seu trabalho?					
18.Trabalhar com clientes deixa-o sem energia?					
19.Sente que dá mais do que recebe quando trabalha com clientes?*					

Depression, anxiety, and stress scale - DASS-21 (Apóstolo et al., 2006; Lovibond & Lovibond, 1995)

Por favor, leia as seguintes afirmações e assinale o número (0, 1, 2, 3) que indica quanto cada afirmação se aplicar a si durante os últimos dias. Não há respostas corretas ou incorretas. Não demore demasiado tempo em cada resposta.

A escala de classificação é a seguinte:

0- Não se aplicou a mim.

1- Aplicou-se a mim um pouco, ou durante parte do tempo.

2- Aplicou-se bastante a mim, ou durante uma boa parte do tempo.

3 - Aplicou-se muito a mim ou maior parte do tempo.

Nos últimos dias:

Item	0	1	2	3
20.Eu tive dificuldade para me acalmar/descomprimir				
21.Dei-me conta que tinha a boca seca				
22.Não conseguia ter nenhum sentimento positivo				
23.Senti dificuldade em respirar (por exemplo, respiração excessivamente rápida ou falta de respiração na ausência de esforço físico)				

24.Foi-me difícil tomar iniciativa para fazer coisas				
25.Tive tendência para reagir exageradamente em certas situações				
26.Senti tremores (por exemplo, das mãos ou das pernas)				
27.Senti-me muito nervoso				
28.Preocupei-me com situações em que poderia vir a sentir pânico e a fazer papel de ridículo				
29.Senti que não havia nada que me fizesse andar para a frente (ter expectativas positivas)				
30.Senti que estava agitado				
31.Senti dificuldades em relaxar				
32.Senti-me triste e oprimido				
33.Fui intolerante quando qualquer coisa me impedia de realizar o que estava a fazer				
34.Estive perto de entrar em pânico				
35.Não me consegui entusiasmar com nada				
36.Senti que não valia muito como pessoa				
37.Senti que andava muito irritável				
38.Senti o bater do meu coração quando não fazia esforço físico (Ex.: sensação de aumento do bater do coração ou falhas no bater do coração)				
39.Tive medo sem uma boa razão para isso				
40.Senti que a vida não tinha sentido nenhum				

Grupo IV – Questionário Nórdico Musculoesquelético

Instruções para preenchimento:

- Por favor, responda a cada questão, selecionando a caixa apropriada;
- Não deixe nenhuma questão em branco, mesmo se não tiver nenhum problema em qualquer parte do corpo;

1. Considerando os **últimos 12 meses**, teve algum problema (tal como dor, desconforto ou dormência) nas seguintes regiões?

<u>**Nota:**</u> Se sentir dor em alguma das regiões deve selecionar **SIM**. Se não sentir dor deve selecionar **NÃO**

	Sim	Não
**1.** Pescoço		
**2.1** Ombro direito		
**2.2** Ombro esquerdo		
**3.1** Cotovelo direito		
**3.2** Cotovelo esquerdo		
**4.1** Punho / Mão direitos		
**4.2** Punho / Mão esquerdos		
**5.** Região torácica		
**6.** Região lombar		

**7.** Ancas / Coxas		
**8.** Joelhos		
**9.** Tornozelo / Pés		

2. Responda, **apenas,** se tiver algum problema

	2.Durante os **últimos 12 meses** teve que evitar as suas atividades normais (trabalho, serviço doméstico ou passatempos) por causa de problemas nas seguintes regiões:		3.Teve algum problema nos **últimos 7 dias**, nas seguintes regiões:	
	**Sim**	**Não**	**Sim**	**Não**
**1.** Pescoço				
**2.1** Ombro direito				
**2.2** Ombro esquerdo				
**3.1** Cotovelo direito				
**3.2** Cotovelo esquerdo				
**4.1** Punho / Mão direitos				
**4.2** Punho / Mão esquerdos				
**5.** Região torácica				
**6.** Região lombar				
**7.** Ancas / Coxas				
**8.** Joelhos				
**9.** Tornozelo / Pés				

4. Selecione o **nível de dor que sente para cada região**, em que:

0 – Sem dor | 10 – Dor máxima:

	**0**	**1**	**2**	**3**	**4**	**5**	**6**	**7**	**8**	**9**	**10**
**1.** Pescoço											
**2.1** Ombro direito											
**2.2** Ombro esquerdo											
**3.1** Cotovelo direito											
**3.2** Cotovelo esquerdo											
**4.1** Punho / Mão direitos											
**4.2** Punho / Mão esquerdos											
**5.** Região torácica											
**6.** Região lombar											
**7.** Ancas / Coxas											
**8.** Joelhos											
**9.** Tornozelo / Pés											

ErgoBus: Prevenção de LMERT em motoristas de autocarros

Grupo V

1. **Observações/Sugestões de Melhoria:**

Muito obrigada pela sua colaboração!

Email: tania4teixeira@hotmail.com

Tânia Silva
